# The effect of health education intervention on the home management of malaria among the caregivers of children aged under 5 years in Ogun State, Nigeria

**DOI:** 10.1186/2047-783X-17-11

**Published:** 2012-05-17

**Authors:** Kehinde O Fatungase, Olorunfemi E Amoran, Kabir O Alausa

**Affiliations:** 1 Department of Community Medicine and Primary Care, College of Health Sciences, Olabisi Onabanjo University Teaching Hospital, Sagamu, Nigeria; 2 Department of Community Medicine and Primary Care, Olabisi Onabanjo University Teaching Hospital, Sagamu, Nigeria

**Keywords:** Home management, Malaria, Health education intervention, Children aged under 5 years, Nigeria

## Abstract

**Background:**

Malaria is currently the most important cause of death and disability in children aged under 5 years in Africa. A health education interventional study of this nature is essential in primary control of an endemic communicable disease such as malaria. This study was therefore designed to determine the effect of health education on the home management of Malaria among the caregivers of children under 5 years old in Ogun State, Nigeria.

**Methods:**

The study design was a quasi-experimental study carried out in Ijebu North Local Government Area of Ogun State. A multistage random sampling technique was used in choosing the required samples for this study and a semi-structured questionnaire was used to collect relevant information. The intervention consisted of a structured educational program based on a course content adapted from the national malaria control program. A total of 400 respondents were recruited into the study, with 200 each in both the experimental and control groups, and were followed up for a period of 3 months when the knowledge and uptake of insecticide treated net was reassessed.

**Results:**

There was no statistically significant differences observed between the experimental and control groups in terms of sociodemographic characteristics such as age (*P* = 0.99), marital status (*P* = 0.48), religion (*P* = 0.1), and income (*P* = 0.51). The majority in both the experimental (75.0%) and control (71.5%) groups use arthemisinin-based combination therapy as first line home treatment drugs pre intervention. Post health education intervention, the degree of change in the knowledge of referral signs and symptoms in the experimental group was 52.8% (*P* < 0.0001) while it was 0.2% in the control group (*P* = 0.93). Tepid sponging improved by 45.0%, paracetamol use by 55.3%, and the use of herbs and other drugs were not significantly influenced in the experimental (*P* = 0.65 and 0.99) and control group (*P* = 0.89 and 0.88), respectively. Furthermore, there was a 55.7% (*P* = 0.001) increase in the proportion of respondents using the correct dose of arthemisinin-based combination therapy in the home management of malaria and 23.9% (*P* < 0.001) in the proportion using it for the required time.

**Conclusions:**

The study concludes that there is a shift in the home management of malaria with the use of current and effective antimalarial drugs. It also demonstrated the effect of health education on the promptness of appropriate actions taken among the respondents for early diagnosis and treatment. Early diagnosis and appropriate treatment can be guaranteed if caregivers are knowledgeable on prompt actions to be taken in the home management of malaria.

## Background

The vast majority of malaria deaths occur in Africa, south of the Sahara, where malaria also presents major obstacles to social and economic development. Malaria has been estimated to cost Africa more than US$12 billion every year in lost GDP, even though it could be controlled for a fraction of that sum [1]. Malaria is Africa’s leading cause of under-5 mortality (20%) and constitutes 10% of the continent’s overall disease burden [2]. One of the greatest challenges facing Africa in the fight against malaria is early diagnosis and treatment of malaria before it becomes complicated. This relates to all aspects of health behavior especially at the household level including home management of diseases and self-medication. Resistance to chloroquine, the cheapest and most widely used antimalarial drug, is common throughout Africa because of inappropriate and incorrect use, particularly in the southern and eastern parts of the continent [2,3]. Resistance to sulfadoxine-pyrimethamine (SP), often seen as the first and least expensive alternative to chloroquine, is also increasing in east and southern Africa. As a result of these trends, many countries have to change their treatment policies and use drugs which are more expensive, including combinations of drugs, which it is hoped will slow the development of resistance.

Malaria is the most prevalent parasitic endemic disease which is preventable, treatable, and curable. Yet it remains one of the major health problems in Africa [4,5]. The malaria situation is deteriorating despite numerous interventions that have been instituted so far. The obstacles to the success of these interventions are socio-cultural, economic, and political in nature [3]. Malaria is currently the most important cause of death and disability in children aged under 5 years in Africa [5]. Modern medicine has tended to interpret health in terms of medical interventions, and to overemphasize the importance of medical technology. It is important to promote the concept of health as the result of the interaction of human beings and their total environment. The World Health Organization (WHO) advocates the combined approach of ITNs and EDT in its Roll Back Malaria initiative [6-8]. A control strategy comprising proper application of existing means was encouraged; early diagnosis and treatment (EDT) of symptomatic malaria to prevent progression to severe and potentially fatal stages; preventive measures including use of ITNs and selective residual spraying; and prediction, containment and, if possible, prevention of epidemics; and strengthening of local capacities, especially caregivers were recommended [6,7].

In Nigeria, malaria is responsible for 60% of outpatient visits and it is one of the leading causes of under-five mortality, accounting for 30% of total deaths, 25% of infant mortality, and 11% of maternal deaths, with over 90% of the population at risk of malaria [4]. About half of this population will have at least one attack per year and close to 300,000 children die of malaria each year. Over ₦132 billion is estimated as expenditure on malaria annually in form of treatment costs, prevention, and loss of manpower [8]. In Nigeria it accounts for 40% of public health expenditure, 30-50% of inpatient admissions, and up to 50% of outpatient visits in areas with high malaria transmission [2,9].

A health education interventional study of this nature is not only an essential tool in primary control of an endemic communicable disease such as malaria, it also relates to all aspects of health behavior including home management of diseases and self-medication. The efficacy levels of the drugs that were previously used on a wide programmatic basis for the management of uncomplicated malaria have been undermined by the parasite resistance trend observed [2,3]. There has been an increasing antimalarial drug resistance to hitherto first and second line drugs (chloroquine and SP) which has compounded malaria therapy in the country leading to the adoption of artemether/lumefantrine (AL), an artermisinin combination therapy (ACT) as the drug of choice. Artemisinin combined drugs are the recommended mode of treatment of uncomplicated malaria because of its prompt and effective action and quick resolution of the illness. This will reduce the progression of illness to complicated malaria, thereby reducing the malaria disease burden. It will also delay development of resistance to either of the components of the drug.

This study was designed to help mothers improve their personal habits and to make the best use of available first aid treatment for minor ailment. Although health education interventions have been carried out in several study settings [6,10,11] few have considered the effect of multiple interventions on attitude, knowledge, and treatment seeking behavior of mothers of under–5 s. This study was therefore designed to determine the effect of health education on the home management of malaria among the mothers of under-5 s in Ogun State, Nigeria. Primary healthcare as stated in the Alma Ata declaration underscores the importance of health education as one of the key methods of preventing and controlling prevailing health problems. This study seeks to test the effect of this on mothers’ behavior in a rural setting. Effective malaria program involved multiple intervention aimed at disease prevention and control, with an increasing emphasis on health education [12].

## Methods

### The study area

The study was carried out in Ijebu North Local Government Area of Ogun State. Ijebu North Local Government is one of the 20 local governments in Ogun State. The experimental study was carried out in Oru, a semi-urban town in Ijebu North Local Government Area of Ogun State, Nigeria. It is bordered in the east by Iperin, the west by Awa, the north by Ijebu-Igbo, and the south by Ago Iwoye. Oru has a population of about 100,000 people (2006 population census). The control study was carried out in the Atikori ward at Ijebu-Igbo, a semi-urban town in Ijebu North Local Government Areawith a population of about 150,000 people (2006 population census) [13].

The two study areas are inhabited by people of mixed cultural background and the languages are predominantly Ijebu/Yorubas. They are also inhabited by Olabisi Onabanjo University students and workers including lecturers and other non-teaching staff. The people are mostly farmers planting cocoa, cassava, kolanuts, and so on, while some are engaged in small-scale businesses. The local government headquarters are in Ijebu-Igbo at Oke-Sopen. There are seven political wards: three wards, including Oru, are located in the southern axis of the local government, and four wards, including the control study area, are located in the northern axis of the local government. The local government has social infrastructures such as electricity, water supply, and schools (primary, secondary, and tertiary). The health institutions within the local government consist of seven primary healthcare centers and a government general hospital.

There are three primary healthcare centers (PHCs) in the southern axis and four PHCs and a government general hospital located in the northern axis of the local government. Malaria is holo-endemic in this local government, with heavy rainfalls in February and March and July to October every year.

### Study design

The project design was a quasi-experimental study to determine the effect of malaria education program on the mothers’ knowledge about malaria prevention and management of under-5 children. Two political wards, one randomly selected from the southern axis (Ijebu-Oru) and the other one randomly selected from the northern axis (Ijebu-Igbo), formed the experimental and control groups, respectively. It was decided to choose the experimental and control groups from two different ends (north and south axes) of the local government to prevent cross-interference during and after the intervention periods. The distance between the experimental and the control group is about 10 km.

### Theoretical framework

The study was carried out in three phases: pre-intervention, intervention, and post-intervention phases. Phase one (pre-intervention) involved cross-sectional comparative descriptive study, while phase two involved comprehensive health education intervention in the experimental group only. Phase three (post-intervention) involved comparative study between the experimental and control group.

### Pre-intervention activities

These included the following: (1) obtaining official information to proceed with the project from the LGA authorities; (2) consent of the mothers of under-5 children to fully participate at all stages of the project was obtained; (3) fifty households were selected in a nearby community (Ilaporu) for pre-testing of the questionnaire before large-scale study - the questionnaires were pre-tested with the research assistants, who had debriefing on field experiences and proffered solutions to identified problems - amendments were made, which led to re-designing aspects of the instrument that were ambiguous or lacked clarity; (4) a baseline survey to determine the mothers’ knowledge, attitude, and practice (KAP) about malaria prevention and management was conducted using the corrected questionnaires - this represented the pre-training assessment for the intervention group and the initial assessment for the control group - a semi-structured questionnaire was used to collect data and was administered with the assistance of eight selected trained research assistances (community health extension workers); answers to questions on sociodemographic variables, knowledge, attitudes, and practice about malaria prevention and treatment were collected; an average of 20 questionnaires were administered daily for 10 days; the same was also done for the control group; (5) the training curriculum and program was based on course content adapted from the training manual for the management of malaria in Nigeria 2005.

### Intervention activities

The intervention consisted of a structured educational program based on a course content adapted from the national malaria control program and the information obtained from the gaps in knowledge identified from the distributed questionnaire formed the basis of the training. Training sessions were conducted during which various aspects of the management and control of malaria were taught. Multiple health channels were used. These include: a training workshop, use of education materials such as posters, story book, and malaria post signs (Appendix VIII). Two malaria sign posts were erected at the community health center, which is beside the community major market. The sign posts indicated graphic descriptions of the insecticide-treated bed net and directions for its use. The benefits and annotations were written in Yoruba. The sign posts were located at conspicuous positions around the health center, which is not far from the major market. Colorful malaria posters indicating malaria symptoms and signs in children and annotated diagrams for prevention and treatment were pasted at different locations within the health center (Appendix VIII).

Each batch was trained for 1 day. The training consisted of three modular units which were: knowledge about malaria transmission, its prevention and treatment; attitude on malaria prevention strategies; and practice of malaria prevention and treatment practices. Each module consisted of a lecture and an exercise. The training period lasted for 2 weeks with training taking place 5 days a week. The participating mothers/guardians were divided into 10 batches of 20.

Training was held for 5 hours a day from 10:00 to 15:00. The training method was both didactic and participatory.

### Post-intervention

The post-intervention evaluation was carried out to determine a residual gain in malaria-related KAP 3 months after the training and initial assessment in the intervention and control groups, respectively. This represented the 3 months post-training assessment. Evaluation of the effects of training was done using standardized scores for the various variables during analysis.

### Sample size

The minimum sample size needed was obtained from the formula for comparing proportions between two groups.

(1) n=Ζ1−α/22Po1−POΖβPo1−Po+P11−p1Po−P12

The outcome measure for computing the sample size was the proportion of mothers using artemisinin combination drugs in Nigeria using mosquito nets, P1 = 12% (NDHS, 2003).

The study hoped to improve the percentage by 15%.

P2=Minimum proportion of mothers expected to be utilizing mosquito net after the intervention = 27%

P0=average of P1 and P2 = (12 + 27)/2 = 19.5%

Z_1- α/2_=Standard normal deviate corresponding to level of significant (α) of 5% = 1.96

Z_β_=Standard normal deviate corresponding to type II error of 10% (Power = 90%) = 1.28.

D=design effect of 1.5 for the sampling design used

P1-P2=15%

Then

(2) $$n=1.5{\left\{\frac{\left(1.96\;2\times 0.195\left(1-0.195\right)+1.28\;0.12\left(1-0.12\right)+0.27\left(1-0.27\right)\right)}{0.15}\right\}}^{2}$$

The minimum sample size from the above formula is 182 for each group. However 200 women per group were studied after allowing for a 10% attrition rate.

### Subject selection

Inclusion criteria were as follows: only mothers or guardians who are permanent residents (resident in the area >6 months) and currently having children <5 years old living with them were included in the study.

Exclusion criteria were as follows: mothers or guardians whose children <5 years old were not living with them at the time of the study were not included in the study.

### Sampling technique

A multistage random sampling technique was used in choosing the required samples for this study. Ijebu North Local Government has seven political wards. Four of these wards were located in the northern axis of the local government and the remaining three were in the southern axis of the local government. Each of the political wards served as a cluster. The first step was to choose whether the northern part or the southern part became the experimental or control group; this was done by tossing a coin. From the list of political wards in each axis, a ward was selected by simple random sampling technique by casting a lot, for example balloting using same size of papers, thoroughly mixing them up, and then picking one at random. House enumeration was carried out by the researcher and two officials from the town-planning unit of the local government. A total number of 1,800 houses were counted in the experimental and control wards, respectively. A systematic random sampling technique using a sample interval of five and four in the experimental and control wards, respectively, was used to choose 200 houses each in experimental and control groups. The sample interval was obtained by dividing the total number of houses by the sample size in the experimental and control wards, respectively (1000/200 and 800/200). The first house was determined by using the table of random number to pick a house from the house enumeration list and the one household was studied per house and this was randomly selected. In the two groups, a simple random sampling technique was carried out by ballottement to choose a caregiver of an under-5 from a household where there was more than one caregiver with an under-5 in the house. Where there was one caregiver in a house, the caregiver of the under-5 automatically qualified to participate in the study, and in situations where a caregiver has more than one under-5, the youngest child was selected.

### Data collection

A baseline survey to determine the mothers’ knowledge about malaria prevention and management was conducted using the corrected questionnaires (pre-training assessment). A semi-structured questionnaire was used to collect data and was administered with the assistance of eight selected trained research assistants (community health extension workers). Answers to questions on socio-demographic variables, and KAP about malaria prevention and treatment were collected.

The data collectors were trained for 3 days on the study objectives, survey methods, and completion of the questionnaires. The proficiency of the questionnaires and interviewers were verified through pre-testing and the deficiencies were corrected. Furthermore, field monitoring was carried out to check quality of the data being collected. The questionnaire was verbally translated into Yoruba where applicable and translated back into English for validity.

Fifty households were selected in a nearby community (Ilaporu) for pre-testing of the questionnaire before the large-scale study. The questionnaires were pre-tested with the research assistants, who had debriefing on field experiences and proffered solutions to identified problems. Amendments were made, which led to re-designing aspects of the instrument that were ambiguous or lacked clarity.

A training curriculum and program based on the health educational needs was developed and this formed the baseline data collected for the study group survey. The training was carried out in the health center situated in Oru following the approval from the local government authority. A post-training evaluation was done after 3 months on the experimental group to determine the gains in malaria prevention and management-related KAP using the same (self-administered and in some cases assisted) questionnaire, while no intervention was administered to the control group.

### Data analysis

The questionnaires were kept safe and confidential and checked for proper completion on collection from participants. The data were entered into SPSS statistical software version 12. Frequencies were generated for detection of errors (data editing). Data were summarized using means, standard deviation, and proportions.

To measure the effectiveness of health education intervention, the degree of change was measured and this was subjected to the tests of significance (McNemar’s Chi-square, *P* values) where appropriate. The degree of change between two samples was calculated by finding the difference in percentage point between the proportions in the second sample with a given attribute and the proportion in the first sample with the same attribute. This was calculated in both the experimental and control groups.

For the purpose of analysis, marital status was re-categorized as ‘currently married’ and ‘not married’. ‘Not married’ include single, the separated, and the widows. Knowledge of malaria was categorized as ‘good’ and ‘poor’: ‘good’ entailed the knowledge that malaria is caused by mosquito insect while other responses regarding malaria causation were categorized as a ‘poor’ level of knowledge. Knowledge of signs and symptoms of malaria were assessed, with 1 point ascribed to each correct answer. The respondents were then categorized as good, fair, and poor. Scores of 4 to 6 were categorized as good, whereas 3 to 4 were rated fair, and 0 to 2 poor.

### Ethical consideration

The research proposal was approved by the Olabisi Onabanjo University Teaching Hospital Ethical Committee. Informed consent was obtained from the Chairman, Ijebu North Local Government Area, and the community leaders. Oral and written consent was obtained from the selected mothers and guardians before administering the questionnaires. The participants promised to fully cooperate and they were also assured of their freedom to opt out at any stage of the project. The participants/respondents were assured of confidentiality and this assurance was indicated on the questionnaire (non-inclusion of self-identifying characteristics).

## Results

### Socio-demographic characteristics

Four hundred mothers/guardians of children under 5 years of age completed the questionnaire at the commencement of the study. These respondents were divided into two groups: the control and experimental (intervention) groups. The control group had 200 respondents (50% of the total number of participants); 180 (90%) of them were available to complete the questionnaire after the 3-month intervention period. The experimental group had 200 respondents (50% of the total number of participants) of which 190 (95%) responded to the study questionnaires after the 3-month intervention period. The socio-demographic characteristics of the caregiver and the index child in both the experimental and control groups are shown in Tables 1 and 2.

**Table 1:** Socio-demographic characteristics of the respondents

	**Experimental group*n*** **= 200 (%)**	**Control group*n*=200 (%)**	**Test statistic value (*X***^**2**^**)**	***P*value**
*Age (years)<2525 to*	52 (26.0)	53 (26.5)	0.02	0.99
34	105 (52.5)	105 (52.5)		
35	43 (21.5)	42 (21.0)		
**Total**	**200 (100)**	**200 (100)**		
*Marital status*				
Currently married	184 (92.0)	180 (90.0)	0.49	0.48
Others	16 (8.0)	20 (10.0)		
**Total**	**200 (100)**	**200 (100)**		
*Religion*				
Christianity	133 (66.6)	148 (74.0)	2.68	0.1
Islam	67 (33.3)	52 (26.0)		
**Total**	**200 (100)**	**200 (100)**		
*Mother’s income (₦)*				
Less than 2500	66 (33.0)	74 (37.0)	2.33	0.51
2500-4999	64 (32.0)	59 (29.5)		
5000-7499	27 (18.5)	19 (9.5)		
7500 +	43 (21.5)	48 (24.0)		
**Total**	**200 (100)**	200 (100)		
*Father’s Income (₦)*				
<2500	11(5.5)	12(6.0)	1.13	0.77
2500-4999	19(9.5)	23(11.5)		
5000-7499	38(19.0) 132(66.0)	43(21.5) 122(61.0)		
7500 +	132(66.0)	122(61.0)		
**Total**	200 (100)	**200 (100)**		

**Table 2:** Index of children’s characteristics

**Characteristic**	**Experiment**	**Control*n*** **= 200**	**Test statistic**	***P*value**
	***n*** **= 200 [%]**	**[%]**	**value (*X***^**2**^**)**	
*Sex*				
Male	96 (48.0)	98 (49.0)	0.04	0.84
Female	104 (52.0)	102 (51.0)		
*Age group (months*)				
< 6	31 (15.5)	36 (18.0)	6.20	0.10
6-11	37 (18.5)	28 (14.0)		
12-23	57 (28.5)	76 (38.0)		
24-35	44 (22.0)	32 (16.0)		
36+	31 (15.5)	28 (14.0)		
*Person child shares bed with*				
Mother	145 (72.5)	144 (72.0)	0.49	0.78
Parents	49 (24.5)	52 (26.0)		
Other sibling	6 (3.0)	4 (2.0)		

More than half of the respondents fell into the 25-34-year-old age group in both the experimental (52.5%) and control (52.5%) groups, followed by 26.0% (experimental) and 26.5% (control group) in the <25 years category and those >35 years were 21.5% (experimental) and 21.0% (control group). A high percentage of the experimental (92.0%) and control (90.0%) groups were married. Over 66.6% (experimental) and 74.0% (control) were Christians while the rest were Muslims (Table 1). About 40% of the experimental group were earning above ₦5000 compared with 33.5% of the control group. While 52.9% of the experimental group had up to secondary school education, only 55% of the control group had the same level of education, followed by a primary level of education in 29.2% of the experimental group and 25% of the control group, while for those with no formal education, about 5% and 7% were found among the experimental and control groups, respectively. There was no significant statistical differences observed between the experimental and control groups in terms of socio-demographic characteristics such as age (*P* = 0.99), marital status (*P* = 0.48), religion (*P* = 0.1), and income (*P* = 0.51).

### Index of children’s characteristics

The characteristics of the children are shown in Table 2 below. About 38% of the children were between 12 and 23 months, in both the experimental (28.5%) and the control (38.0%) groups, followed by 14.3% aged between 6 to 11 months, 18.5% (experimental) and 14.0% of the (control), while the least was found between the age group 36 + months (15.5% and 14.0% of the experimental and control group, respectively). There were slightly more females in both the experimental (52.0%) and control groups (51.0%). The majority of children in the experimental (72.5%) and control (72.0%) groups share the same bed with their mother, while 24.5% (experimental) and 26.0% (control group) share the same bed with both parents. The remaining children share the same bed with others. There was no statistically significant difference in the characteristics of index child both in the experimental and control groups in terms of sex (*P* = 0.84), age (*P* = 0.10), and the person the child is sharing the bed with (*P* = 0.78).

### Knowledge of signs and symptoms of malaria

The knowledge of signs and symptoms of malaria was statistically significantly improved by health education in the experimental group (*P* < 0.001) while there was no statistically significantly change in the control group (*P* = 0.68). The degree of change for the experimental group in terms of improvement by educational intervention for referral signs and symptoms in the experimental group was 52.8% while it was 0.2% in the control group (*P* = 0.93). This is as shown in Table 3. Similarly, knowledge of good prevention practices also improved by 48.6% (*P* < 0.001) in the experimental group with no significant change in the control group (*P* = 0.72).

**Table 3:** Knowledge scores by signs, symptoms, and prevention of malaria in children

**A. Signs and symptoms of malaria fever in children**								
	**Experimental group**				**Control group**			
	**Pre-intervention*n*** **= 200 (%)**	**Post-intervention*n*** **= 190 (%)**	**Degree of change (%)**	***P*value**	**Pre-intervention*n*** **= 200 (%)**	**Post-intervention*n*** **= 180 (%)**	**Degree of change (%)**	***P*value**
Good	11 (5.5)	116 (61.1)	55.6	<0.001	9 (4.5)	9 (5.0)	0.5	0.66
Fair	150 (75.0)	56 (29.5)	-45.4		166 (83.0)	143 (79.5)	-3.8	
Poor	39 (19.5)	28 (9.4)	-10.3		25 (12.5)	28 (15.5)	3.3	
**Total**								
**B. Knowledge of signs and symptoms of malaria that will need referral (danger signs)**								
	**Experimental group**				**Control group**			
	**Pre-intervention*n*** **= 200 (%)**	**Post-intervention*n*** **= 190 (%)**	**Degree of change (%)**	***P*value**	**Pre-intervention*n*** **= 200 (%)**	**Post-intervention*n*** **= 180 (%)**	**Degree of change (%)**	***P*value**
Good	6 (3.0)	106 (55.8)	52.8	<0.001	6 (3.0)	5 (2.8)	0.2	0.93
Fair	114 (57.0)	70 (36.8)	-20.2		132 (66.0)	116 (65.5)	-0.5	
Poor	80 (40.0)	14 (7.4)	-32.6		62 (31.0)	59 (31.7)	-0.7	
**Total**								
**C. Knowledge of prevention of malaria in children**								
	**Experimental group**				**Control group**			
	**Pre-intervention*n*** **= 200 (%)**	**Post-intervention*n*=190 (%)**	**Degree of change (%)**	***P*value**	**Pre-intervention*n*** **= 200 (%)**	**Post-intervention*n*** **= 180 (%)**	**Degree of change (%)**	***P*value**
Good	6 (3.0)	98 (51.6)	48.6	<0.001	4 (2.0)	4 (2.2)	0.0	0.72
Fair	12 (6.0)	65 (34.2)	28.2		44 (22.0)	46 (25.6)	3.5	
Poor	182 (91.0)	27 (14.2)	-77.2		151 (75.5)	130 (72.2)	-3.5	

### Home management of malaria by caregivers

The majority in both the experimental (75.0%) and control (71.5%) groups use arthemisinin-based combination theraphy (malact@-a combination of Artesunate and Amodiquine) as first line home treatment drugs. This antimalarial drug malact was given free of charge from all the health centers by the local government. However the use of paracetamol (34.0% and 36.0%) and tepid sponging (15.4% and 37.5%) in the experimental and control group, respectively, was low pre-intervention. There was a statistically significant improvement in the experimental group (*P* < 0.001) post-intervention compared with the control group (*P* > 0.5). All of them in the experimental group stopped the use of chloroquine (0.0%) with the majority (97.5%) using malact@ with tepid sponging improving by 45.0% and paracetamol use by 55.3%. There was almost no change in the use of these modes of treatment in the control group. Worthy of note is the fact that the use of herbs and other drugs were not significantly influenced by health education (*P* = 0.65 and 0.99, respectively) in the experimental and control group (*P* = 0.89 and 0.88), respectively, as shown in Tables 4 and 5.

**Table 4:** Home remedy for malaria treatment

	**Experimental group**				**Control group**			
	**Pre- intervention*n*** **= 200 (%)**	**Post- intervention*n*** **= 190 (%)**	**Degree of change (%)**	***P*value**	**Pre- intervention*n*** **= 200 (%)**	**Post- intervention*n*** **= 180 (%)**	**Degree of change (%)**	***P*value**
Malact	150 (75.0)	172 (90.5)	15.4	<0.001	140 (70.0)	71.5	1.2	0.81
Paracetamol	68 (34.0)	170 (89.5)	55.3	< 0.001	70 (35.0)	36.3	1.2	0.81
Chloroquine	30 (15.0)	0 (0.0)	-15.4	< 0.001	40 (20.0)	21.2	1.2	0.81
Tepid sponging	78 (39.0)	160 (84.2)	45.0	< 0.001	74 (37.0)	37.8	0.3	0.96
Herbal Medicine	20 (10.0)	13 (6.8)	-3.2%	0.65	22 (11.0)	11.9	0.8%	0.89
Other drugs	16 (8.0)	15 (7.9)	-0.1%	0.99	18 (9.2)	10.1	0.9	0.88

**Table 5:** Malaria treatment practices using Malact@ (Artesunate and Amodiquine)

	**Experimental group**				**Control group**			
	**Pre-intervention*n*** **= 200 (%)**	**Post-intervention*n*** **= 190 (%)**	**Degree of change (%)**	***P*value**	**Pre-intervention*n*** **= 200 (%)**	**Post-intervention*n*** **= 180 (%)**	**Degree of change (%)**	***P*value**
*Malact@ efficacy*								
Efficient	195 (97.5)	190 (100.0)	2.5	0.28	194 (97.0)	176 (97.7)	−0.7	0.64
Not efficient	5 (2.5)	0 (0.0)	-2.5		6 (3.0)	4 (2.3)	0.7	
*Malact@ Dose*								
Correct	36 [18.0]	140 [73.7]	55.7%	<0.001	45 [22.5]	40 [22.2]	−0.3%	0.78
Over dose	92 [46.0]	32 [16.8]	29.2%		86 [43.0]	72 [40.0]	− 3.0%	
Under dose	72 [36.0]	18 [9.5]	26.5%		69 [34.5]	68 [37.8]	3.2%	
*Treatment duration (days)*								
1	17 (8.5)	0 (0.0)	−8.5	<0.001	11 (5.5)	10 (5.5)	0.0	0.79
2	26 (13.0)	27 (14.2)	0.9		49 (24.5)	46 (25.6)	0.9	
> 3	120 (60.0)	159 (83.7)	23.9		132 (66.0)	113 (62.8)	-3.2	
3	37 (18.5)	4 (2.1)	-16.3		8 (4.0)	11 (7.1)	3.1	
*Commence treatment after symptom recognition*								
First day	146 (73.0)	185 (97.4)	24.4	<0.001	101 (50.5)	88 (48.9)	−1.6	0.64
Second day	47 (23.5)	5 (2.6)	-20.9		72 (36.0)	72 (40.0)	4.4	
Third day	7 (3.5)	0 (0.0)	-3.5		27 (13.5)	20 (11.1)	-2.6	

### Multiple response

#### Promptness and appropriateness of actions taken by caregivers

Almost all respondents (97.5%) believe in the efficacy of malact@ pre-intervention but only 18.1% and 22.6% in the experimental and control groups, respectively, give the correct dose to their children. Post-intervention, there was 55.7% (*P* = 0.001) increase in the proportion of respondents using the correct dose compared to nil change in the control group (*P* = 0.78). Only 59.5% and 62.7% in the experimental and control groups, respectively, used the drugs for the correct length of time pre-intervention. Post-intervention there was a statistically significant increase of 23.9% (*P* = 0.001) in the proportion using it for the required time with no significant increase in the control group (*P* = 0.79). Furthermore, 72.9% and 50.8% of respondents in the experimental and control group, respectively, commenced treatment at the right time (first day of fever). There was a significant increase of 24.6% (*P* = 0.001) post-intervention in the experimental group with no significant change in the control (*P* = 0.64).This is shown in Table 5.

## Discussion

The fact that the study shows a shift in the home management of malaria with the use of current and effective antimalarial drugs and a reduction in reliance on herbs for the home management of malaria may be attributable to increase in awareness of management of malaria and the free distribution of these antimalarial drugs by the National Malaria Control Programme. Several studies conducted in Nigeria earlier had reported that chloroquine was the commonest antimalarial drug given in the study area [14-16]. Others reported the use of paracetamol as the commonest drug [3,8,17]. This may signify the success of the implementation of the Roll Back Malaria program in the country, thus further indicating that the control and eventual eradication of malaria may be possible with the free distribution of this antimalarial drugs and appropriate health education of caregivers. Combination therapies that include artemisinin derivatives are preferred for being highly effective and also eliminating gametocytes (the sexual forms responsible for transmission of the parasite) [18-21]. However, given the economic constraints of malaria endemic communities in Africa, these communities will not be able to afford more costly artemisinin-based combinations without aid. With these economic restrictions, sustenance of this free distribution may be crucial to the control of malaria disease in this region.

The study shows that overdose of malact® was prescribed by 46.6% of respondents in the experimental group and 48.3% in the control group. Under dosage was given by 38.1% in the experimental group and 28% in the control group. The danger with overdose is that it exposes the children to the toxic effects of malact®, while the under dosage of malact® leads to the development of resistant strains of plasmodium falciparum, and may push simple malaria case to severe form of malaria with all its attendant consequences. This observation was also made by other workers in similar studies [22-26]. At the post-intervention phase 74.6% of the respondents got the correct dose of malact® for children which gave a degree of change of 59.3%. There was a significant association between training and the increased ability of the respondents to get the correct dose of malact®. No such relationship existed in the control group that was not exposed to training. This may indicate that appropriate health education of caregivers may be the key to prevention of the development of resistance strain and this may be crucial to the control and eventual eradication of malaria in Africa.

This study indicate that more than one-quarter of caregivers do not start treatment of child using antimalarial drugs at the appropriate time even when they recognize the onset of malaria. And the training program carried out by the authors had a significant impact on the ability of caregivers to recognize appropriate signs and symptoms for prompt treatment and referral signs for presentation at health centers. Several reports had indicated a high malaria burden in sub-Saharan Africa [2,4,27]. One of the major problems responsible for this may be the inability of the caregiver to recognize when to take action. The authors recommend that a systematic health education program to caregivers should be a component of the Roll Back Malaria program in Africa. Early diagnosis and prompt treatment is essential to control of malaria and this can only be effectively carried out by those at the frontline of care at home.

The study strongly demonstrated the effect of health education in the home management of malaria. There was a statistically significant relationship between the proportions of appropriate actions taken among the respondents in the experimental groups when compared with the control group. A greater proportions of respondents performed tepid sponging (84.2%), gave malact® (90.4%), and gave paracetamol (89.5 %). There was little or no change in the distribution of activities undertaken by mothers before and after the intervention program in the control group. The objective of health education is to make people value health as a worthwhile asset and to show them what they can do as individuals, families, and communities to improve their own health [9]. The more African people value health the more they will be willing to make the appropriate allocation of resources to promote and safeguard their own health. The community will be more prepared to allocate resources for improvement of environmental sanitation, and for other priorities within the health services in the control and eventual eradication of malaria.

Given the nature of the experimental study, the interpretation of the study results should be done with caution. The study might also have been faced with a lot of influence from external forces which might have introduced bias into the study. The prevention of the cross-over effect could not be totally guaranteed between the experimental and control groups during and after the intervention program.

## Conclusions

The study concludes that there is a shift in the home management of malaria with the use of current and effective antimalarial drugs and reduction in reliance on herbs attributable to increase in awareness of management of malaria and the free distribution of these antimalarial drugs by the National Malaria Control Programme. It also demonstrated the effect of health education on the proportions and promptness of appropriate actions taken among the respondents for early diagnosis and treatment. Early diagnosis, appropriate treatment, and prompt referral can be guaranteed if caregivers are knowledgeable on prompt actions to be taken at home for effective management of malaria.

## Competing interests

The authors declare that they have no competing interests.

## Authors’ contributions

FOK conceived the study and participated in its design, AOE participated in the analysis and helped to draft the manuscript, AOK participated in the coordination. All authors read and approved the final manuscript.
